# Dysregulated thyroid hormones correlate with anxiety and depression risk in patients with autoimmune disease

**DOI:** 10.1002/jcla.23573

**Published:** 2020-11-18

**Authors:** Xiaorong Wu, Kaikai Zhang, Yulong Xing, Wei Zhou, Yanqiu Shao, Guizheng Li, Qing Rui

**Affiliations:** ^1^ Department of Rheumatology Gaochun Branch Nanjing Drum Tower Hospital The Affiliated Hospital of Nanjing University Medical School Nanjing China

**Keywords:** anxiety, autoimmune disease, depression, HADS, thyroid hormones

## Abstract

**Background:**

Autoimmune disease (AID) patients always present with increased risk of psychiatric disorders, and thyroid function or thyroid hormone may play a critical role in the development of anxiety and depression. Thus, this study aimed to assess the free triiodothyronine (FT3), free tetraiodothyronine (FT4), thyroid‐stimulating hormone (TSH) levels, and their correlations with anxiety/depression in patients with AID.

**Methods:**

Ninety‐eight AID patients and 100 health controls (HCs) were recruited. Serum samples were obtained from all the participants to detect FT3, FT4, and TSH levels. Anxiety and depression were determined using the HADS assessment.

**Results:**

HADS‐Anxiety score, anxiety subject percentage, HADS‐Depression score, and depression subject proportion were elevated in AID patients compared with HCs. FT3 and FT4 were downregulated while TSH was upregulated in AID patients compared with HCs. In AID patients, FT3 and FT4 negatively correlated with HADS‐Anxiety score, and they were downregulated in patients with anxiety compared to patients without anxiety. Meanwhile, FT3 and FT4 were negatively associated while TSH level positively associated with HADS‐Depression score. Besides, FT3 and FT4 reduced, but TSH level was of no difference in patients with depression compared to patients without depression. Additionally, increased FT4 independently correlated with both reduced anxiety risk and depression risk.

**Conclusions:**

FT3, FT4, and TSH are dysregulated, and FT4 has the potential to serve as an independent biomarker related to anxiety as well as depression in AID patients. These findings may provide some information on the values of thyroid hormones in facilitating the management of AID patients with anxiety/depression.

## Graphical abstract

**Background:** This study aimed to assess the free triiodothyronine (FT3), free tetraiodothyronine (FT4), thyroid‐stimulating hormone (TSH) levels, and their correlations with anxiety/depression in patients with autoimmune disease (AID). **Methods:** Ninety‐eight AID patients and 100 health controls (HCs) were recruited. Then serum samples were obtained from all the participants to detect FT3, FT4, and TSH levels. Anxiety and depression were determined using the Hospital Anxiety and Depression Scale (HADS) assessment. **Results:** HADS‐Anxiety (HADS‐A) score (*P* < .001), anxiety subject percentage (*P* = .002), HADS‐Depression (HADS‐D) score (*P* < .001), and depression subject proportion (*P* < .001) were notably elevated in AID patients compared to HCs. Most interestingly, FT3 (*P* < .001) and FT4 (*P* < .001) levels were downregulated while TSH level (*P* < .001) was upregulated in AID patients compared to HCs. In AID patients, FT3 (*P* = .004) and FT4 (*P* = .001) levels were negatively correlated with HADS‐A score, and they were downregulated in patients with anxiety compared to patients without anxiety (*P* = .025 and *P* < .001, respectively). Meanwhile, FT3 (*P* = .010) and FT4 (*P* < .001) levels were negatively associated while TSH level (*P* = .041) was positively associated with HADS‐D score. Besides, FT3 (*P* = .003) and FT4 (*P* < .001) levels were reduced, but TSH level was of no difference in patients with depression compared to patients without depression. Multivariate logistic regression analysis revealed that increased FT4 level was independently correlated with both reduced anxiety risk (*P* = .001) and depression risk (*P* < .001). **Conclusions:** FT3, FT4, and TSH are dysregulated, and FT4 has the potential to serve as an independent biomarker related to anxiety as well as depression in AID patients.

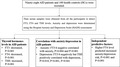

## 1 INTRODUCTION

Autoimmune disease (AID), a self‐attacking disease caused by aberrant immune reactions from immune cells to autoimmune antibodies, affects approximately 10% of the worldwide population with an increased incidence in females.^1^, ^2^ AID can be organ specific or systemic which affects multiple organs, more importantly, both types are destructive due to that patients must bear with large physical burden and face an increased risk of disability.^3^, ^4^ Treatment of AID nowadays is predominantly based on systemic immune suppression; however, the life expectancy and disability of patients still need improvement. Moreover, AID not only destroys the organs and tissues, but also interferes with the mental health in patients, for example, systemic lupus erythematosus (SLE), multiple sclerosis (MS), and Crohn's disease patients all present with increased risk of psychiatric disorders.^5^, ^6^, ^7^

Nowadays, the gravity of psychiatric disorders in patients with AID has been increasingly mentioned, and studies report that patients with AID present with a higher risk of developing anxiety and depression.^8^, ^9^, ^10^ Moreover, anxiety and depression are factors that impair not only the quality of life but also recovery of AID, for instance, the existence of anxiety or depression in patients with myasthenia gravis may lead to a misdiagnosis of a disease progression, which is due to that symptoms like fatigue is common in both anxiety/depression and myasthenia gravis.^11^ However, mechanism of the psychiatric disorders development in AID patients is still mysterious, indicating that more effort is needed in this area. Recently, more and more studies suggest that dysregulated thyroid function may be involved in the pathogenesis of anxiety and depression, such as hypothyroidism is reported to correlate with increased anxiety and depression severity, and the TSH level is elevated in patients with anxiety/depression compared to healthy subjects.^12^, ^13^ These data all indicate that thyroid function or thyroid hormone may play a critical role in the development of anxiety and depression. Based on these findings, and thyroid dysfunction as well as anxiety/depression are both common in AID, we presumed that the levels of thyroid hormones may associate with the presence of anxiety or depression in AID. Nonetheless, to our best knowledge, association between thyroid hormones and anxiety/depression is still obscure in AID patients.

Herein, to explore the potential value of thyroid hormone levels for assisting in the management of AID patients with anxiety and/or depression, the aim of this study was to assess the levels of FT3, FT4, TSH, and their correlations with anxiety/depression in patients with AID.

## 2 MATERIALS AND METHODS

### 2.1 Participants

This study consecutively recruited 98 AID patients treated in our hospital between January 2019 and December 2019. The inclusion criteria were as follows: (a) clinically diagnosed as AID, including sicca syndrome (SS), rheumatoid arthritis (RA), systemic lupus erythematosus (SLE), ankylosing spondylitis (AS), inflammatory myopathy (IM), etc; (b) age within 18 ~ 80 years; and (c) able to independently complete the assessment of anxiety and depression using Hospital Anxiety and Depression Scale (HADS). The exclusion criteria included (a) patients complicated with serious endocrine disease, hematological malignancies, thyroid cancer, or other solid tumors; (b) patients who presented with organic damage of heart, liver, or kidney; (c) patients who had a history of severe mental illness or cognitive impairment; and (d) patients who were pregnant or lactating women. Additionally, a total of 100 healthy subjects who underwent health examination in our hospital from January 2020 to March 2020 were enrolled as healthy controls (HCs) in the current study. All HCs were required to have matched age and gender with AID patients, no obvious abnormalities in physical examination and be able to fulfill the HADS assessment, where the matching was performed by controlling a gender ratio of 1:2 (female/male) and a age range of 45 ~ 60 years. This study was approved by Institutional Review Board of our hospital, and all participants provided written informed consents.

### 2.2 Data and sample collection

Characteristics of participants were recorded after enrollment, which included age, gender, education duration, marry status, and employment status. Using vacuum blood collection tube, 5 mL fasting venous blood was extracted from each participant. After natural coagulation, the serum was centrifugalized at 825 *g* and stored in a refrigerator at −70°C for future use.

### 2.3 FT3, FT4, and TSH detection

The FT3, FT4, and TSH levels in serum of participants were determined by radioimmunoassay (RIA) method using commercial FT3 RIA kit, FT4 RIA kit, and TSH RIA kit (Beijing North Institute of Biotechnology Co., Ltd., China). All procedures of RIA were conducted according to the handbooks of kits, and all tests were in triplicate.

### 2.4 Anxiety and depression evaluation

After enrollment, guidance about HADS assessment was provided to all participants, and then, participants were required to independently fulfill the HADS for evaluation of anxiety and depression. HADS was originally developed as a psychometric instrument to identify depression and generalized anxiety in medical patients, which comprised of two subscales: HADS‐Anxiety subscale (HADS‐A) and HADS‐Depression (HADS‐D) subscale. Both the HADS‐A subscale and the HADS‐D subscale contained seven items which were scored from 0 to 3 points individually, resulting in 0‐21 points totally for each subscale. The HADS‐A score > 7 was defined as anxiety; accordingly, the HADS‐D score > 7 was defined as depression.^14^

### 2.5 Statistical analysis

Data analysis was performed using SPSS 22.0 statistical software (IBM, USA), and figure plotting was carried out using GraphPad Prism 7.02 (GraphPad Software Inc, USA). Continuous data were expressed as mean with standard deviation (M ± SD), and categorical data were presented as count with percentage (No. (%)). Difference of continuous data between two groups was determined by Student's *t* test, and difference of categorical data was determined by chi‐square test. Correlation analysis was determined by Pearson's test. Factors associated with anxiety/depression of AID patients were assessed by univariate and forward stepwise multivariate logistic regression model analyses. *P* value < .05 was considered as statistically significant.

## 3 RESULTS

### 3.1 Characteristics of HCs and AID patients

Mean age was 53.4 ± 4.0 years in HCs and was 54.4 ± 11.8 years in AID patients (*P* = .416) (Table 1). The numbers of male and female were 33 (33.0%) and 67 (67.0%) in the HCs and were 37 (37.8%) as well as 61 (62.2%) in the AID patients (*P* = .484). Mean education duration was 7.9 ± 5.1 years and 8.7 ± 4.6 years in HCs and AID patients, respectively (*P* = .296). In HCs, there were 16 (16.0%) who were single/divorced/widowed and 84 (84.0%) who were married; in AID patients, there were 23 (23.5%) who were single/divorced/widowed and 75 (76.5%) who were married (*P* = .186). The numbers of employed and unemployed subjects were 81 (81.0%) and 19 (19.0%) in HCs, and were 69 (70.4%) as well as 29 (29.6%) in AID patients (*P* = .082). In addition, in AID patients, the numbers of patients who had SS, RA, SLE, AS, IM, and other AID were 35 (35.7%), 20 (20.4%), 16 (16.3%), 8 (8.2%), 6 (6.1%), and 13 (13.3%), respectively. In brief, no difference in characteristics was found between the HCs and AID patients.

**Table 1:** Characteristics of AID patients and HCs

Items	HCs (N = 100)	AID patients (N = 98)	*P* value
Age (y), M ± SD	53.4 ± 4.0	54.4 ± 11.8	.416
Gender, No. (%)			.484
Male	33 (33.0)	37 (37.8)	
Female	67 (67.0)	61 (62.2)	
Education duration (y), M ± SD	7.9 ± 5.1	8.7 ± 4.6	.296
Marry status, No. (%)			.186
Single/divorced/widowed	16 (16.0)	23 (23.5)	
Married	84 (84.0)	75 (76.5)	
Employment status, No. (%)			.082
Employed	81 (81.0)	69 (70.4)	
Unemployed	19 (19.0)	29 (29.6)	
Disease type, No. (%)			‐
SS	‐	35 (35.7)	
RA	‐	20 (20.4)	
SLE	‐	16 (16.3)	
AS	‐	8 (8.2)	
IM	‐	6 (6.1)	
Other AID	‐	13 (13.3)	

Abbreviations: AID, autoimmune disease; AS, ankylosing spondylitis; HCs, heath controls; IM, inflammatory myopathy; M ± SD, mean ± standard deviation; RA, rheumatoid arthritis; SLE, systemic lupus erythematosus; SS, sicca syndrome.

### 3.2 Comparison of FT3, FT4, and TSH levels between HCs and AID patients

**Figure 1:**
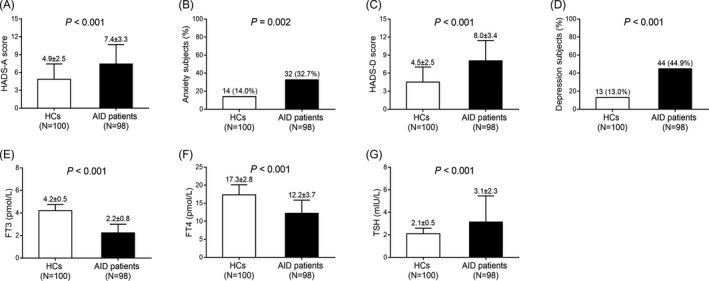
Anxiety, depression, FT3, FT4, and TSH in AID patients and HCs. HADS‐A score (A), anxiety subjects (B), HADS‐D score (C), depression subjects (D), FT3 level (E), FT4 level (F), and TSH level (G) between AID patients and HCs. AID, autoimmune disease; HCs, healthy controls; HADS‐A, Hospital Anxiety and Depression Scale‐Anxiety subscale; HADS‐D, Hospital Anxiety and Depression Scale‐Depression subscale; FT3, free triiodothyronine; FT4, free tetraiodothyronine; TSH, thyroid‐stimulating hormone

The HADS‐A score (*P* < .001) (Figure 1A), anxiety subject percentage (*P* = .002) (Figure 1B), HADS‐D (*P* < .001) (Figure 1C) score, and depression subject proportion (*P* < .001) (Figure 1D) were elevated in AID patients compared with HCs. Primarily, the FT3 (*P* < .001) (Figure 1E) and FT4 (*P* < .001) (Figure 1F) levels were markedly decreased, while the TSH (*P* < .001) level (Figure 1G) was notably increased in AID patients compared with HCs.

### 3.3 Associations of FT3, FT4, and TSH levels with anxiety in AID patients

**Figure 2:**
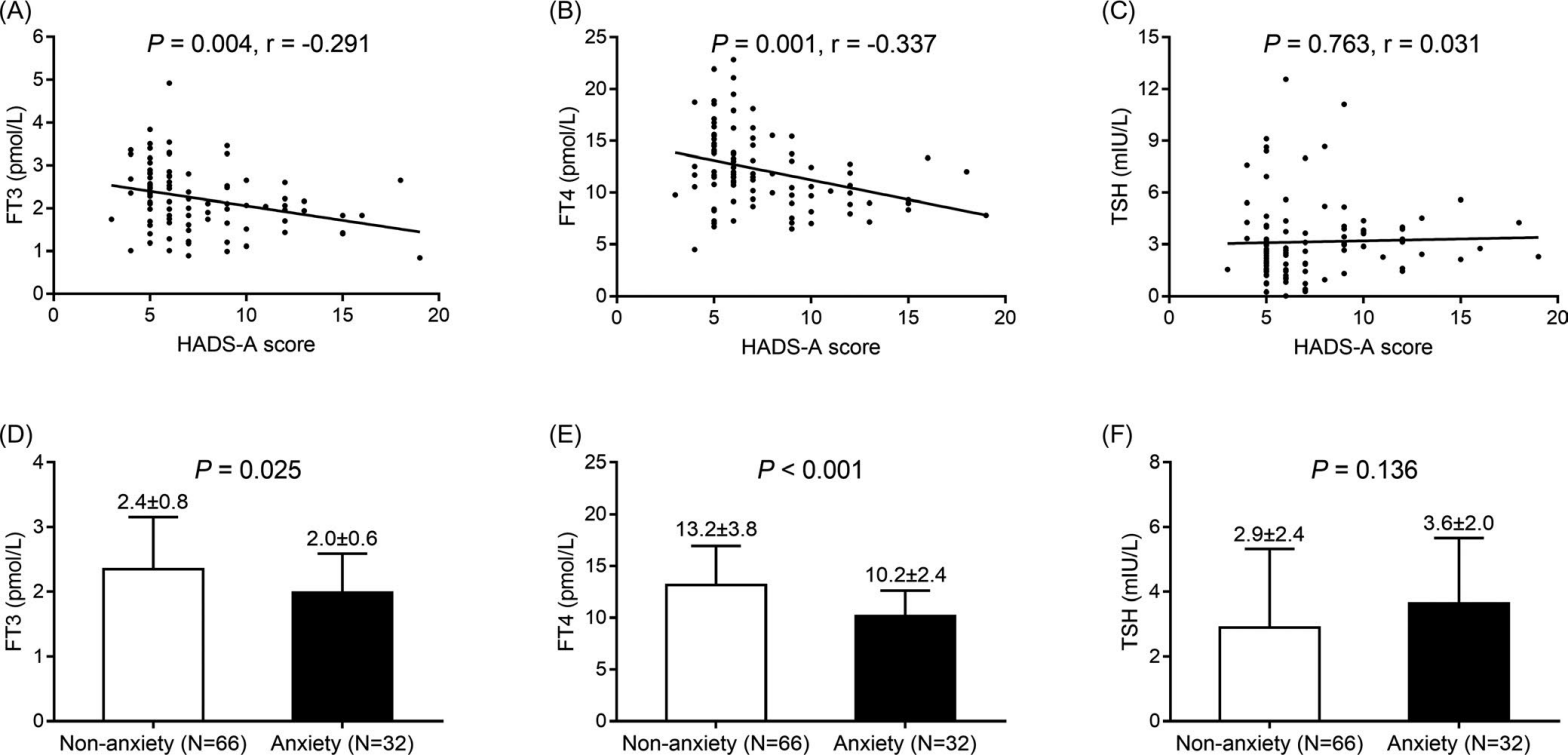
Correlations of FT3, FT4, and TSH with anxiety in AID patients. Associations of FT3 level (A), FT4 level (B), and TSH level (C) with HADS‐A score, and the FT3 level (D), FT4 level (E), as well as TSH level (F) between AID patients with anxiety and AID patients with no anxiety. FT3, free triiodothyronine; FT4, free tetraiodothyronine; TSH, thyroid‐stimulating hormone; HADS‐A, Hospital Anxiety and Depression Scale‐Anxiety subscale; AID, autoimmune disease

The FT3 (*P* = .004) (Figure 2A) and TF4 (*P* = .001) (Figure 2B) levels were negatively correlated, while TSH (*P* = .763) (Figure 2C) level was not correlated with HADS‐A score in AID patients. In addition, the FT3 (*P* = .025) (Figure 2D) and FT4 (*P* < .001) (Figure 2E) levels were downregulated while the TSH (*P* = .136) (Figure 2F) level did not vary in AID patients with anxiety compared to AID patients without anxiety. These all indicated negative correlations of FT3 and FT4 with anxiety in AID patients.

### 3.4 Associations of FT3, FT4, and TSH with depression in AID patients

**Figure 3:**
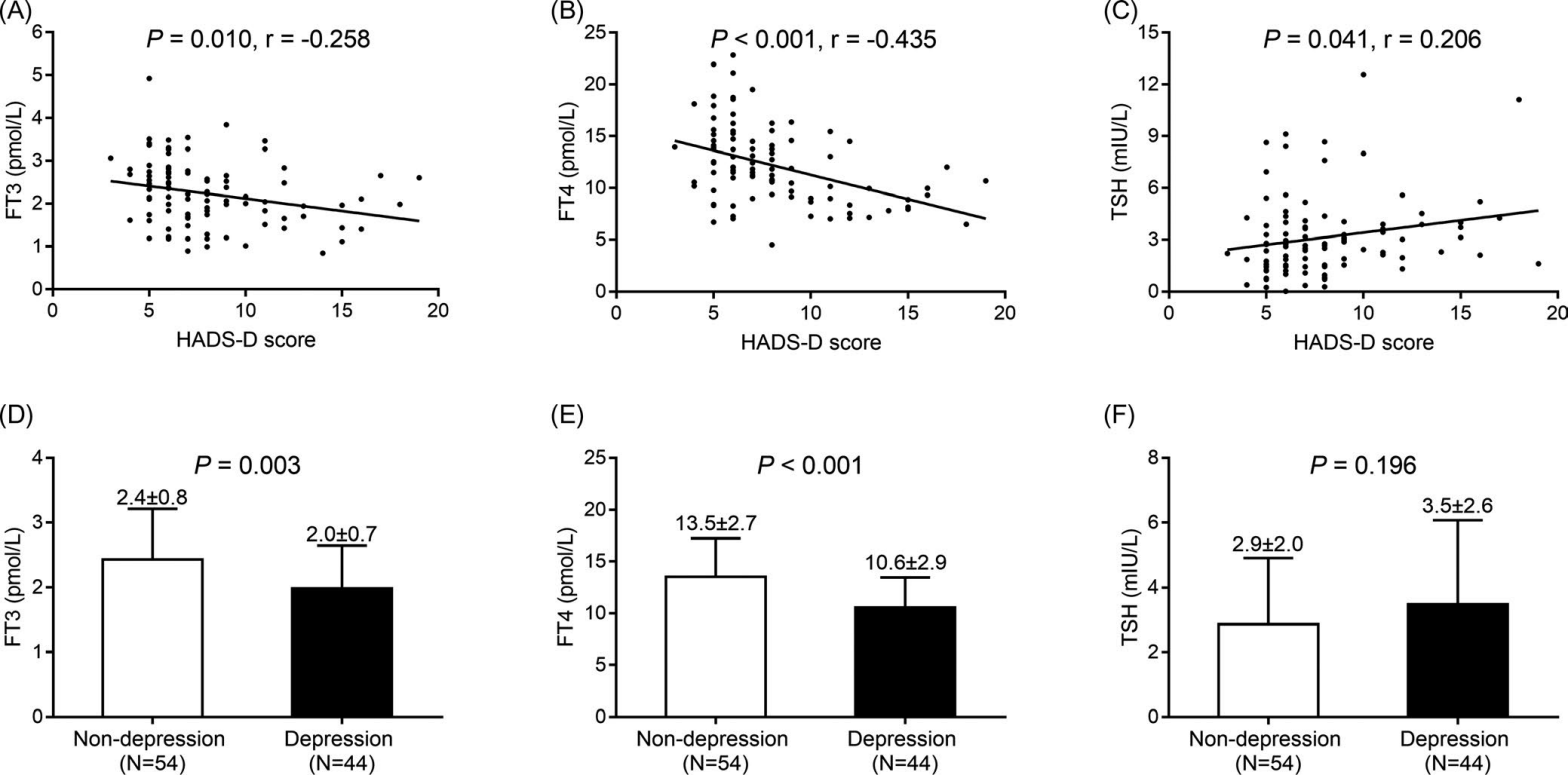
Correlations of FT3, FT4, and TSH with depression in AID patients. Correlations of FT3 level (A), FT4 level (B), and TSH level (C) with HADS‐D score, and the FT3 level (D), FT4 level (E) as well as TSH level (F) between AID patients with depression and AID patients with no depression. FT3, free triiodothyronine; FT4, free tetraiodothyronine; TSH, thyroid‐stimulating hormone; HADS‐A, Hospital Anxiety and Depression Scale‐Depression subscale; AID, autoimmune disease

The levels of FT3 (*P* = .010) (Figure 3A) and FT4 (*P* < .001) (Figure 3B) were negatively associated while TSH level (*P* = .041) (Figure 3C) was positively associated with HADS‐D score in AID patients. Besides, the FT3 (*P* = .003) (Figure 3D) and FT4 (*P* < .001) (Figure 3E) levels were reduced but the TSH (*P* = .196) (Figure 3F) level was of no difference in AID patients with depression compared with AID patients without depression. These data suggested that FT3 and FT4 were negatively associated with depression in AID patients.

### 3.5 Analysis of factors that affected anxiety in AID patients

In univariate logistic regression, increased FT3 (*P* = .028) and FT4 (*P* < .001) levels were correlated with decreased anxiety risk in AID patients; in addition, gender female (*P* = .009) correlated with increased anxiety risk in AID patients (Table 2). Furthermore, in forward stepwise multivariate logistic regression, increased FT4 (*P* = .001) level was independently correlated with reduced risk of anxiety, while gender female (*P* = .018) was independently correlated with higher risk of anxiety in AID patients.

**Table 2:** Factors associated with anxiety in AID patients

Items	Logistic regression model			
	*P* value	OR	95% CI	
			Lower	Higher
Univariate logistic regression				
FT3	.028	0.496	0.266	0.927
FT4	<.001	0.746	0.635	0.878
TSH	.143	1.144	0.956	1.368
Age	.135	1.029	0.991	1.069
Gender (Female)	.009	3.838	1.397	10.546
Education duration	.224	0.950	0.874	1.032
Marry status (Married)	.209	0.540	0.206	1.413
Employment status (Employed)	.235	0.578	0.234	1.427
Disease type				
Other AID	Reference	‐	‐	‐
SS	.923	1.067	0.289	3.938
RA	.251	0.400	0.084	1.913
SLE	.685	0.727	0.156	3.386
AS	.223	0.229	0.021	2.456
IM	.637	1.600	0.227	11.266
Forward stepwise multivariate logistic regression				
FT4	.001	0.746	0.629	0.884
Gender (Female)	.018	3.684	1.250	10.859

Abbreviations: AID, autoimmune disease; AS, ankylosing spondylitis; FT3, free triiodothyronine; FT4, free tetraiodothyronine; IM, inflammatory myopathy; RA, rheumatoid arthritis; SLE, systemic lupus erythematosus; SS, sicca syndrome; TSH, thyroid‐stimulating hormone.

### 3.6 Analysis of factors that affected depression

As to factors that affected depression in AID patients, the univariate logistic regression analysis revealed that increased FT3 (*P* = .005) and FT4 (*P* < .001) levels were predictors for decreased risk of depression in AID patients; besides, gender female (*P* = .020) predicted increased depression risk while status of married (*P* = .029) predicted reduced depression risk in AID patients (Table 3). Then, the forward stepwise multivariate logistic regression analysis disclosed that increased FT4 level (*P* < .001) was an independent predictive factor for reduced depression in AID patients; however, gender female (*P* = .046) independently predicted increased depression risk.

**Table 3:** Factors associated with depression in AID patients

Items	Logistic regression model			
	*P* value	OR	95% CI	
			Lower	Higher
Univariate logistic regression				
FT3	.005	0.420	0.229	0.771
FT4	<.001	0.761	0.657	0.881
TSH	.200	1.123	0.940	1.342
Age	.151	1.026	0.991	1.062
Gender (Female)	.020	2.786	1.171	6.625
Education duration	.247	0.955	0.882	1.033
Marital status (Married)	.029	0.336	0.127	0.892
Employment status (Employed)	.079	0.454	0.188	1.097
Disease type				
Other AID	Reference	‐	‐	‐
SS	.208	2.382	0.617	9.204
RA	.591	1.500	0.342	6.583
SLE	.705	1.350	0.286	6.379
AS	.751	1.350	0.211	8.617
IM	.053	11.250	0.972	130.221
Forward stepwise multivariate logistic regression				
FT4	<.001	0.763	0.656	0.887
Gender (Female)	.046	2.604	1.018	6.663

Abbreviations: AID, autoimmune disease; AS, ankylosing spondylitis; FT3, free triiodothyronine; FT4, free tetraiodothyronine; IM, inflammatory myopathy; RA, rheumatoid arthritis; SLE, systemic lupus erythematosus; SS, sicca syndrome; TSH, thyroid‐stimulating hormone.

## 4 DISCUSSION

Previous literatures have suggested intimate correlations of anxiety and depression with AID. A prior study illuminates that the prevalence of depression and anxiety is markedly increased in SLE patients compared to healthy subjects.^9^ Another study conducted in patients with RA reveals that there are 32.4% patients presenting with depression as assessed by Korean version of the Beck Depression Inventory‐second edition.^15^ In addition, a study reports that there are 65% of patients with RA who are diagnosed as depression, and 37.5% of them have moderate or severe depression; furthermore, this study also reveals that depression and anxiety are associated with function damage and pain caused by arthritis.^16^ Partially in consistent with the previous studies, we found in our study that in patients with different AIDs, the prevalence of anxiety and depression was elevated compared with control healthy subjects. With regard to reasons to these results, we presumed that it might include the following possibilities: first, patients with AID normally bear with more psychological stress compared with healthy subjects, mostly due to the disease burden or the reduction of quality of life caused by the disease, such as inactivity, fatigue, and disfiguring. Second, previous researches reveal that anxiety/depression and AID may share similar pathological mechanism, which is the interaction between central nervous system and immune system. Briefly, the disorder in immune system may result in dysregulations in central nervous system, which finally lead to anxiety and/or depression.^17^, ^18^

AID is often accompanied by dysregulation in thyroid function, which is common in several AIDs; for instance, SLE and RA patients are more likely to obtain thyroid dysfunction.^19^, ^20^, ^21^, ^22^ Such as, a large‐scale cross‐sectional study elucidates that hypothyroidism prevalence is notably higher in patients with RA compared with age‐ and gender‐matched healthy subjects (16.0% vs 11.7%).^21^ Another case‐control study reveals that the titer of triiodothyronine (T3) is decreased in SLE patients than that in age‐ and gender‐matched healthy subjects (125.2 ± 35.6 vs 136.2 ± 26.5).^23^ Besides, a cohort study illustrates that in patients with RA, the prevalence of hypothyroidism and subclinical hypothyroidism are 12% and 11%, respectively; and RA patients with treated hypothyroidism/subclinical hypothyroidism have decreased quantitative insulin sensitivity check index (QUICKI) score and increased homeostasis model assessment for insulin resistance (HOMA‐IR) score (which indicate insulin resistance) compared to RA patients with adequate thyroid function.^24^ These studies all indicate that thyroid dysfunction is more frequent in AID patients compared with general population, and most of the cases are hypothyroidism. In our study, we found that the FT3 and FT4 levels were reduced while TSH level was increased in AID patients compared with healthy subjects, these results may be derived from that: On one hand, some of the AID affect multiple systems in human body that also include the thyroid gland, which might result in a dysregulated FT3, FT4, and TSH levels in AID patients.^25^, ^26^ On the other hand, thyroid gland is more sensitive to autoimmune change compared to other organs, and the thyroid cells can secrete various factors that are immunological to interact with the immune system, which might eventually cause a dysregulation in thyroid hormones levels in patients with AID.^27^

More importantly, thyroid dysfunction is also involved in the etiology of anxiety and depression. For instance, a previous study reveals that adult mice with deficiency of Type 2 Deiodinase, which indicates a lack of thyroid hormones, present with elevated anxiety behaviors and fear memory compared with the healthy mice.^28^ Besides, another study elucidates that untreated hypothyroidism is correlated with an increased score of Beck depression inventory (BDI‐II), and untreated hyperthyroidism is associated with diagnosis of major depression disorder (MDD) in 2142 individuals.^12^ Additionally, a study reveals that in Chronic Hepatitis B (CHB) patients, the mean level of FT3 and percentage of patients with decreased FT3 level (less than < 3.5 mol/L) are markedly increased in CHB patients with clinical and subclinical depression compared to CHB patients without depression.^29^ These data unveil that thyroid dysfunction is correlated with higher risk of developing anxiety and depression. However, the associations between these indexes related to thyroid function and anxiety or depression in AID patients are seldom investigated. In our study, we found strong and negative correlations of FT3 and FT4 levels with anxiety and depression, besides a relatively weak correlation between TSH level and depression; moreover, higher FT4 level was an independent predicting factor for decreased risk of both anxiety and depression in AID patients. And these findings may be derived from that: First, thyroid dysfunction has long been indicated in the pathogenesis of mood disorders including anxiety and depression, and thyroid dysfunction is frequent in AID, which subsequently contributes to a positive correlation between FT3/FT4 level and anxiety/depression in AID patients.^30^ Second, there are studies revealing that anti‐thyroid antibodies are involved in the development of depression, which could also provide some explanation.^31^ In addition, we also found that gender was an independent predictive factor for both anxiety and depression in patients with AID, which may be related to the correlation between gender and AID severity or the development of anxiety/depression in AID patients. For instance, it is reported that female gender is correlated with increased disease severity in patients with myasthenia gravis.^32^ Moreover, a study elucidates that in patients with SLE, female patients present with increased symptoms of anxiety and depression compared to the male patients.^33^ However, precise mechanism of the correlation between gender and anxiety/depression in patients with AID should be evaluated in the future.

There were several limitations in the present study. First, the sample size of 98 AID patients was relatively small, which could result in a less strong statistical power. Second, the long‐term predicting values of FT3, FT4, and TSH levels for anxiety as well as depression were not assessed, since the follow‐up time was relatively short in our study. Third, other scales for assessing anxiety and depression may be needed in our study to validate the results of anxiety/depression prevalence in AID patients. Fourth, apart from education duration, marry status, and employment status, other factors possibly correlated with anxiety and depression should be included in the analysis in our study. However, it was too difficult to obtain the information about all the possible anxiety/depression related factors of all the subjects of our study in the clinical practice. In the future, a validation cohort is needed to provide more evidence of the results in this study.

In summary, FT3, FT4, and TSH are dysregulated, and FT4 has the potential to serve as an independent biomarker for predicting anxiety as well as depression in AID patients.
